# 178. Assessment of an IOTA (Intravenous to Oral Transition of Antimicrobial Therapy) in Gram Negative Bacteremias at a Community Teaching Hospital

**DOI:** 10.1093/ofid/ofad500.251

**Published:** 2023-11-27

**Authors:** Victoria Wilson, Sara R Gardner, Beth Cady

**Affiliations:** HSHS St. John's Hospital, Chatham, Illinois; Southern Illinois University Edwardsville School of Pharmacy, Chatham, Illinois; Southern Illinois University at Edwardsville School of Pharmacy, Hillsboro, Illinois

## Abstract

**Background:**

Retrospective studies suggest that IV to oral therapy for gram-negative bacteremia has similar outcomes to IV regimens. The benefits of transitioning patients from IV to oral antibiotic therapy are well-recognized. The purpose of this study was to assess the percentage of patients (with a gram-negative bacteremia from a urinary source) who were transitioned from IV to oral antibiotics based on a pre-determined checklist.

**Methods:**

This retrospective cohort study was performed at a 400-bed hospital from January 1, 2019 to May 31, 2022 . The primary objective was to assess the percentage of patients with a gram negative bacteremia (from a urinary source) who were eligible and switched from IV to oral antibiotics. Patients were included in the study if they were > 18 years old, had a positive blood culture for *E. coli* or *K. pneumoniae* (with > 1 susceptible oral antibiotic), had a monomicrobial infection from a urinary source, signs of clinical improvement, could tolerate oral medication, and had at least 24 hours of IV therapy.

**Results:**

Sixty patients out of 200 (30%) with a gram negative bacteremia met the above eligibility criteria to be switched from IV to oral therapy. Sixty three percent (38/60) were switched to oral antibiotics for the completion of therapy. A majority of patients (95%) had *E. coli* bacteremia. Of the eligible patients, 70% (42/60) had received an ID consultation. Of those, 62% (26/42) were switched. Among the 18 eligible patients who did not receive an ID consult, 67% were switched to PO. Reasons for not switching (as determined from the EHR) for the 22 eligible patients include: 3 patients had pathogens resistant to fluoroquinolones, 8 had contraindications (unclear), 3 had concomitant infections, 7 had reasons unknown, and 1 patient denied oral antibiotics.

**Conclusion:**

Most patients (63%) who met inclusion criteria derived by previous studies were switched from IV to oral antibiotics in the presence of monomicrobial *E. coli* or *Klebsiella pneumoniae* bacteremia from a urinary source with clinical improvement. There is an opportunity to educate providers to transition more patients from IV to oral antibiotics with a gram negative bacteremia.

**Disclosures:**

**All Authors**: No reported disclosures

